# Ready for change? The effect of profession and organization on patient safety culture and organizational readiness for change – a cross-sectional study

**DOI:** 10.1186/s12913-026-14187-w

**Published:** 2026-02-18

**Authors:** Anna Ivert, Sara Freyland, Olof Stephansson, Frida Viirman, Malin Edqvist

**Affiliations:** 1 Clinical Epidemiology Division, Department of Medicine, Karolinska Institutet, Solna, Stockholm, Sweden; 2 Department of Women’s and Children’s Health, Karolinska Institutet, Stockholm, Sweden; 3 Department of Women’s Health and Allied Health Professionals, Karolinska University Hospital, Stockholm, Sweden; 4 Department of Women’s and Children’s Health, Uppsala University, Uppsala, Sweden

**Keywords:** Patient safety culture, Organizational readiness, Obstetrics, Midwifery

## Abstract

**Background:**

The aim of this study was to investigate whether profession and organization is associated with patient safety culture and organizational readiness for change prior to implementing a care process aimed at improving communication and teamwork during childbirth.

**Methods:**

A cross-sectional design using a web-based survey sent to midwives, physicians and nurse assistants at seven labor wards in Sweden. The Hospital Survey On Patient Safety (HSOPS 2.0) covering ten dimensions of patient safety culture and the short version of the Swedish Organizational Readiness for Change scale (ORC-SWE-S) covering seven dimensions were used. A two-way MANOVA was conducted to assess the effects of profession and organization, and their interaction. Subsequently, a two-way ANOVA was performed to explore group-level differences by profession, labor ward, and their interaction. The relationships between HSOPS 2.0 and ORC-SWE-S dimensions were assessed using Pearson’s correlation analysis.

**Results:**

In total 645 participants responded, yielding a response rate of 75.4%. The results of the MANOVA revealed significant differences across professions, labor wards, and their interaction for both the HSOPS 2.0 (Wilk’s Λ = 0.82–0.29, *p* ≤ 0.03) and ORC-Swe-S dimensions (Pillai’s trace = 0.64–0.41, *p* < 0.001). The two-way ANOVA showed statistically significant differences across professions and labor wards for both HSOPS 2.0 and ORC-SWE-S dimensions. While effect sizes were generally small by profession (η² < 0.05), *Staffing and Work Pace* and *Inadequate Staffing Resources* showed substantial variation by labor ward (η² = 0.43 and 0.40, respectively). Among the safety dimensions, midwives rated *Teamwork* highest, whereas physicians rated *Staffing and Work Pace* lowest. Significant interaction effects between profession and labor ward were observed in multiple dimensions. Pearson’s correlation showed strong positive correlations between *Organizational Trust* and *Organizational Learning* (*r* = 0.55), *Leader Support for Patient Safety* (*r* = 0.56), and *Communication about Error* (*r* = 0.53).

**Conclusion:**

Both profession and organization influenced staff perceptions of patient safety culture and readiness for change, with organization having the greatest impact. Teamwork received the highest ratings, while Staffing and Resource related dimensions varied most across labor wards. The findings underscore the importance of organizational context when implementing new practices.

**Supplementary Information:**

The online version contains supplementary material available at 10.1186/s12913-026-14187-w.

## Background

Ensuring patient safety constitutes a fundamental principle at the core of the healthcare system [1]. Although childbirth is generally considered safe in high income countries, the intrapartum period remains critical for both women and their newborns, as the risk of morbidity increases considerably in the event of complications [2]. Patient safety within maternity care therefore aims to improve the quality of care and reduce the occurrence of preventable adverse events [3]. Such events in intrapartum care may relate to failures to detect fetal distress [4] or to prevent maternal complications, for example delays in the management of postpartum hemorrhage [5]. Sweden is known for having good to excellent outcomes for women and their infants [6, 7]. However, according to the Swedish Health and Social Care Inspectorate (IVO), obstetrics and gynecology is one of the areas within somatic specialist care that receive the highest number of patient complaints. Adverse events reported in maternity care range from 4 to 12%, with estimates suggesting that 50–74% of these events may be preventable [8–10]. Furthermore, considerable and consistent variability in outcomes and interventions across both Swedish regions and hospitals has been reported [11, 12]. Variations in quality of care may therefore be due to workplace i.e., organizational culture. Patient safety culture, a component of organizational culture [13], encompasses the shared beliefs, values, attitudes, norms, and behaviors of employees, influencing how staff perceive and engage with patient safety within their organization [13, 14]. Measuring patient safety culture is important for identifying system-related issues, evaluating targeted interventions, and supporting continuous improvement in patient safety efforts. The Hospital Survey on Patient Safety Culture (HSOPS 2.0) is commonly used for this purpose, capturing key dimensions such as teamwork, open communication, error reporting, and organizational learning to highlight both strengths and areas needing improvement [14].

Despite the fact that most adverse events in maternity care result from failures in communication and teamwork [15], few interventions have been specifically designed to support these aspects in everyday clinical practice. This oversight is significant as many adverse outcomes in intrapartum care develop gradually over time, offering several opportunities for the team to intervene [16]. TeamBirth is a care process designed to improve communication and teamwork among care providers, women, and their partners during childbirth [17]. It involves brief team meetings or “huddles” facilitated by a shared planning board placed in the birthing room to promote transparent and reliable communication. The board displays team members’ names, the woman’s birth preferences, labor progress, the care plan for the woman and fetus, and the timing of the next huddle. Core principles of TeamBirth include recognizing the birthing person and their partner as active members of the care team, reflecting the growing recognition of the relationship between patient safety and person-centered care [18]. The Swedish adapted version, TeamBirth-SWE, was developed, tested, and piloted at two labor wards in Stockholm during 2021–2022. The adapted Swedish version aligns with the SBAR communication tool [19], with board sections corresponding to background and assessment. TeamBirth is used for all women in labor, regardless of risk assessment; consequently, the frequency and duration of huddles, as well as the professional roles involved, vary according to multiple factors, including parity, labor duration, and the presence of chronic conditions or intrapartum complications. The professionals participating in TeamBirth and its huddles are those involved in providing care to women during childbirth. In Swedish maternity care, this relates to midwives, obstetricians, and nurse assistants. Swedish midwives have primary responsibility for intrapartum care regardless of risk status, whereas obstetricians hold medical responsibility for women with chronic conditions and when complications develop during labor or birth [20]. Obstetricians perform operative births and are the final decision-makers regarding cesarean section  [21]. Midwives and obstetricians are supported by nurse assistants, who assist with practical tasks and provide direct care [20]. Most obstetric units in Sweden have an educational mandate and provide clinical training for residents and midwifery students. In many obstetric units, staffing arrangements include a senior coordinating midwife on each shift, with specific responsibility and involvement in acute situations. Altogether, TeamBirth can be considered a complex intervention [22], as it engages all professions involved in childbirth, includes the birthing woman and her partner, and facilitates bedside care planning and decision-making, requiring behavioral change across multiple interacting components [23].

When major initiatives alter how work is conducted and affect all professions involved in care, it is important to examine the context, including organizational conditions that may facilitate or hinder successful implementation. Context encompasses the dynamic and diverse set of factors that may either facilitate or hinder implementation efforts [24]. Implementation science has progressed towards increased use of theoretical approaches to provide better understanding and explanation of how and why implementation succeeds or fails [25]. Organizational readiness for change (ORC) is a key concept in implementation science, often regarded as the essential bridge connecting intentions, active participation, and the results seen when introducing new practices [26]. As a multidimensional concept, ORC has been defined as “organizational members’ psychological and behavioral preparedness to implement change” [27]. ORC further aligns conceptually with the Consolidated Framework for Implementation Research (CFIR) [24, 28], particularly with regard to the *inner setting* domain [29], which encompasses constructs related to readiness such as facilities, resources, workplace culture, and communication. Several scales have been developed to measure ORC [30] including the Organizational Readiness for Change scale [31]. This scale measures dimensions important for change, such as employees’ trust in the organization, staffing resources, workplace satisfaction, challenges related to the work environment, facilities, and whether there is an external demand for change. Since ORC can be understood as part of organizational culture, reflecting how shared values, norms, and experiences shape collective willingness and capacity to implement change it can also be related to patient safety culture [13]. The dimensions of ORC overlap with both enabling factors of patient safety culture and enacted safety behaviors, including leadership commitment, resources, trust, and communication [32]. Assessing and addressing an organization´s readiness for change before introducing interventions that affect staff is therefore of importance. Without readiness, even well-designed changes can fail due to resistance or lack of engagement. Ensuring readiness may reduce resistance, align resources, support staff well-being, and foster a sense of ownership among employees, all of which are key to successful and sustainable implementation [27, 30].

This study is part of a larger project evaluating the implementation and effectiveness of the TeamBirth care process in Sweden. The intervention is hypothesized to improve interprofessional teamwork, communication, information sharing, and patient involvement during childbirth, thereby contributing to improvements in interprofessional collaboration, patient involvement, and patient safety. The larger project is registered at ClinicalTrials.gov, no. NCT06926504, on April 13, 2025.

Against this background, a better understanding of the organizational context in which TeamBirth-SWE will be implemented is needed, particularly in relation to patient safety culture and ORC. Measuring organizational readiness for change and patient safety culture at baseline is of importance for interpreting subsequent implementation outcomes, as these measures may provide insight into conditions within maternity care that facilitate or hinder adoption, adherence, and sustainability. We conjecture that higher readiness for change may strengthen staff engagement and capacity to adopt and adhere to the core components of TeamBirth, while a positive patient safety culture may facilitate consistent use and thereby contribute to sustainability. For the current study we hypothesized that both profession and organization (i.e., workplace) would influence perception of patient safety culture and ORC. The aim of the study was therefore to examine whether professional role and organizational context were associated with patient safety culture and organizational readiness for change prior to implementation of the TeamBirth care process.

## Methods

This study employs a cross-sectional design using a web-based survey. Seven labor wards participate in the Swedish TeamBirth project, representing a variety of settings and geographical locations, labor volume, and teaching statuses (Table 1). The larger project comprises national registry data and a longitudinal survey design with multiple measurement points. For the purpose of the current study, the first measurement at baseline before implementation was used. Eligible to participate were midwives, physicians, and nurse assistants working with intrapartum care at the participating labor wards, including those with hourly employment exceeding 20% over the past year. Exclusion criteria were being a manager or working less than 20% in intrapartum care.

**Table 1:** Characteristics of the participating labor wards

Obstetric unit	No of births 2023	Teaching status	Special Commission	Organizational factors	Outcomes related to safe care for the mother and infant 2023*
Region Southern Sweden Labor ward A	4691	University teaching hospital		Midwives not required to rotate on all shifts Residents do their placement at the labor ward either Lund or Malmö	Unplanned cesarean section: 12% Instrumental birth: 6.7% Severe perineal trauma: 2.1% Apgar < 7 at 5 min: 0.9%
Region Southern Sweden Labor ward B	3299	University teaching hospital	Preterm births from gw 22 + 0	Midwives required to rotate all hours Residents do their placement at the labor ward either Lund or Malmö	Unplanned cesarean section: 13% Instrumental birth: 4.1% Severe perineal trauma: 2.6% Apgar < 7 at 5 min:1.3%
Region Central Sweden Labor ward C	3885	University teaching hospital	Preterm births from gw 22 + 0	Midwives not required to rotate on all shifts	Unplanned cesarean section: 12% Instrumental birth: 6.5% Severe perineal trauma: 3.2% Apgar < 7 at 5 min:1.7%
Region West-Central Sweden Labor ward D	2505	County hospital		Midwives not required to rotate on all shifts	Unplanned cesarean section: 12% Instrumental birth: 4.8% Severe perineal trauma: 3.1% Apgar < 7 at 5 min: 1.6%
Region Southwestern Sweden Labor ward E	1796	County hospital		Midwives not required to rotate on all shifts	Unplanned cesarean section: 13% Instrumental birth: 4.8% Severe perineal trauma: 2.2% Apgar < 7 at 5 min: 1.0%
Region Southwestern Sweden Labor ward F	2105	County hospital		Midwives not required to rotate on all shifts	Unplanned cesarean section: 9.4% Instrumental birth: 4.7% Severe perineal trauma: 2.1% Apgar < 7 at 5 min: 0.7%
Region North-Central Labor ward G	1156	County hospital		Midwives not required to rotate on all shifts	Unplanned cesarean section: 12% Instrumental birth: 6.3% Severe perineal trauma: 5.1% Apgar < 7 at 5 min: 1.6%

Note: Data collected from the Swedish Pregnancy Register 2023

^*^The percentages represent the total number of births conducted at the specified location

Severe perineal trauma: any rupture involving the anal sphincter, ICD codes O70.2, O70.3 and O70.4

The data collection for the baseline survey lasted from September 6, 2023, to October 14, 2024, since the labor wards entered the project at different time periods (Figure S1). The survey was distributed electronically via email, and several reminders were sent to increase participation. All participants were informed about the entire project and the purpose of the baseline survey. Questions included information on participants’ background demographics, along with three validated scales assessing patient safety culture [14, 33], ORC [34], and interprofessional collaboration [35]. Only the scales related to patient safety culture and ORC were used in this study (Supplementary Material Table S1, S2).

### Scales to measure patient safety culture and organizational readiness for change

Patient safety culture was assessed using the *Hospital Survey on Patient Safety Culture* (HSOPS 2.0) [14, 33]. The HSOPS scale was developed by the Agency for Healthcare Research and Quality (AHRQ) to assess patient safety culture within hospitals [33] and updated in 2019. HSOPS 2.0 consists of 34 items that measure patient safety culture across ten dimensions and two single items (Supplementary Material Table S1). *Teamwork* addresses collaboration needed for safe care; while *Staffing and Work Pace* relate to workload, the use of temporary employees, and the work pace. *Organizational Learning – Continuous Improvement* focuses on identifying patient safety issues and implementing changes to enhance safety. *Response to Error* describes learning from mistakes, as well as the lack of support and the possible tendency to blame individuals following safety errors. The dimension *Leader Support for Patient Safety* evaluates how management handles patient safety concerns, whereas *Communication About Error* examines the clinic’s actions when errors occur. *Communication Openness* highlights the willingness to speak up if unsafe situations occur. *Reporting Patient Safety Events* investigates how often safety incidents are reported. *Hospital Management Support for Patient Safety* reflects the extent to which hospital management prioritizes patient safety, and *Handoffs and Information Exchange* addresses failures in communication and information transfer between colleagues and units.

The items are rated on a five-point Likert scale ranging from 1 (“strongly disagree”) to 5 (“strongly agree”) or 1 (“never”) to 5 (“always”), including a response option for “does not apply/don’t know”. Dimension scores were calculated as mean scores across items within each dimension with higher scores indicating stronger agreement. Two single items are included in the scale: (1) *In the past 12 months*,* how many patient safety events have you reported?* and: (2) *How would you rate your unit/work area on patient safety?* with the response options: *Poor*,* Fair*,* Good*,* Very Good*,* Excellent.*

The AHRQ reports internal consistency for the dimensions with Cronbach’s alpha values ranging from 0.67 to 0.89 [14].

ORC was assessed using the shortened and modified Swedish version of the ORC scale (ORC-SWE-S), which is currently being validated from the original ORC-SWE [34]. While the original ORC scale and ORC-SWE comprise 115 items assessing 18 dimensions of organizational readiness, the ORC-SWE-S includes seven dimensions with 33 items (Supplementary Material Table S2); *Organizational Trust*,* Inadequate Staffing Resources*,* Workplace Satisfaction*,* Role model*,* Staff and Workplace Challenges*,* Facilities and Equipment*,* and External demand for Change. Organizational Trust* examines management’s trust in employees and the organization’s willingness to embrace change. *Inadequate Staffing Resources* highlights challenges related to workload, stress, and staffing shortages, while *Workplace Satisfaction* addresses factors such as appreciation, joy, and overall job satisfaction. *Role Model* focuses on individual leadership qualities and expertise, whereas *Staff and Workplace Challenges* encompasses the need for support in improving communication and professional relationships. The sixth dimension *Facilities and Equipment* reflect satisfaction with IT systems and technical resources. Finally, *External Demand for Change* captures pressures for change at various system levels, i.e., from politicians, patients, or the hospital management. The scale uses a five-point Likert scale ranging from 1 (“strongly disagree”) to 5 (“strongly agree”). Dimension scores are calculated as the mean of item responses within each dimension. For the ORC-SWE-S dimensions Organizational Trust, Workplace Satisfaction, Role Model, and Facilities and Equipment, higher scores indicate stronger agreement. For the dimensions Inadequate Staffing Resources, Staff and Workplace Challenges, and External Demand for Change, higher scores indicate stronger disagreement.

The demographic variables included type of profession, age, gender, hospital of employment, working hours, type of employment, percentage of fulltime, working hours per week, and if working clinically at the labor ward (yes/no) (Supplementary Material, Table S3). The continuous variable “percentage of fulltime employment” was dichotomized to full time (100%), yes/no. The variable “physician” was categorized to include both specialists in obstetrics and/or gynecology and residents. The remaining variables that were recategorized included: age (< 25 years, 25–35, 36–45, 46–55, and > 55 years) into < 36 years, 36–45 years, and > 45 years; years of experience in maternity care (< 6 months, 6–11 months, 1–2 years, 3–5 years, 6–10 years, 11–20 years, and ≥ 20 years) into < 3 years, 3–5 years, 6–10 years, and > 10 years; and length of employment at the clinic (< 6 months, 6–11 months, 1–2 years, 3–5 years, 6–10 years, 11–20 years, and ≥ 20 years) into < 1 year, 1–5 years, 6–10 years, and > 11 years.

### Statistical analysis

Descriptive statistics for all background variables were calculated for the overall population as well as by profession, with categorical variables presented as frequencies and percentages. Missing data were handled using a complete case approach, with analyses restricted to participants who had provided responses to the variables required for each analysis. When calculating the HSOPS 2.0 dimensions, responses of “does not apply/do not know” were treated as missing and excluded from the calculation of mean scores. Mean scores for each dimension, as well as Cronbach’s alpha values for HSOPS 2.0 and ORC-SWE-S, were subsequently calculated. The analytical approach was chosen to align with the study aim and to retain the full variability of the ORC-SWE-S and HSOPS 2.0 dimensions [36]. The HSOPS 2.0 and ORC-SWE-S dimensions were stratified by profession and labor ward, and visualized in spider diagrams (Supplementary Material, Figure S2, S3, S4).

The main analysis was conducted separately for the ORC- SWE-S and HSOPS 2.0 scales. A two-way MANOVA was considered appropriate given the presence of multiple correlated outcome dimensions and the factorial study design, allowing examination of the effects of profession and labor ward, as well as their interaction, on the ORC-SWE-S and HSOPS 2.0 dimensions. Distributional characteristics of the data were assessed using skewness and kurtosis statistics and multivariate normality was assumed under the central limit theorem. Homogeneity of covariance matrices was tested using Box’s M test (significance level: 0.01). Multivariate effects are reported using Wilks’ lambda or Pillai’s trace, as appropriate, with Pillai’s trace preferred when greater robustness to assumption violations was required.

To explore how associations varied across different dimensions by profession, labor ward, and their interaction, a two-way ANOVA was performed. This approach reflects the assumption that associations between profession and organization may vary across the different dimensions of HSOPS 2.0 and ORC-SWE-S. For dimensions with statistically significant associations, Tukey’s HSD post-hoc tests were applied to identify specific group differences. Eta squared (η²) was calculated to assess the effect size of associations. According to Cohen’s guidelines, effect sizes were categorized as follows: small (η² = 0.01), moderate (η² = 0.06), and large (η² = 0.14) [37].

Additionally, Pearson’s correlation coefficient was calculated to examine the relationships between the dimensions of the ORC-SWE-S and HSOPS 2.0 scales. The strength of the correlation was interpreted according to Cohen’s guidelines: small correlation (*r* = 0.1), medium correlation (*r* = 0.3), and large correlation (*r* > 0.5) [37]. A significance level of 0.05 was used for all analyses, except for Box’s M test, where a significance level of 0.01 was applied. All analyses were conducted in Stata 18.5 (StataCorp. 2023. *Stata Statistical Software: Release 18*. College Station, TX: StataCorp LLC.).

## Results

A total of 855 surveys were sent to midwives, physicians, and nurse assistants across the seven labor wards, of which 671 were completed. After excluding 26 participants with no direct contact with birthing women, the final sample included 645 participants, yielding a 75.4% response rate. The majority of midwives, physicians, and nurse assistants had permanent positions at their respective clinics. Among the physicians, 46.5% were residents, 34.8% were obstetricians, and 18.1% were gynecologists. Notably, more physicians worked full-time (86.9%) compared to only 49.3% of the midwives and 50.3% of the nurse assistants. Furthermore, a higher proportion of physicians (86.9%) worked rotational shifts covering all hours, compared to 30.4% of midwives and 30.0% of nurse assistants (Table 2). Only midwives in Labor ward B are required to rotate through all shifts (Table 1).

**Table 2:** Overview of participants

	Midwives *n* = 312	Physicians *n* = 153	Nurse assistants *n* = 180
	*n* (%)	*n* (%)	*n* (%)
Age (years)			
<36	77 (24.7)	62 (40.5)	57 (31.7)
36–45	117 (37.5)	60 (39.2)	49 (27.2)
>45	118 (37.8)	31 (20.3)	74 (41.1)
Work experience within intrapartum care (years)			
<3	68 (21.8)	39 (25.5)	68 (37.8)
3–5	61 (19.6)	28 (18.3)	32 (17.8)
6–10	69 (22.1)	32 (20.9)	33 (18.3)
>10	114 (36.5)	54 (35.3)	47 (26.1)
Employment at current labor ward/obstetric unit (years)			
<1	32 (10.3)	23 (15.0)	31 (17.2)
1–5	114 (36.5)	62 (40.5)	69 (38.3)
6–10	74 (23.7)	32 (20.9)	39 (21.7)
>11	92 (29.5)	36 (23.5)	41 (22.8)
Type of employment			
Permanent	300 (96.2)	149 (97.4)	168 (93.3)
Temporary position/Hourly employment	12 (6.7)	4 (2.6)	12 (6.7)
Full-time employment	149 (47.8)	133 (86.9)	89 (49.4)
Missing data	10 (3.2)	0 (0.0)	3 (1.7)
Working hours			
Day shift	156 (50.0)	17 (11.1)	79 (43.9)
Night shift	61 (19.6)	3 (2.0)	47 (26.1)
Rotation all hours	95 (30.4)	133 (86.9)	54 (30.0)

Physicians include obstetricians, gynecologists, and residents

The results of the MANOVA revealed a statistically significant difference in the HSOPS 2.0 dimensions across professions (F(20, 352) = 1.74, *p* = 0.03; Wilk’s Λ = 0.82) and labor wards (F(60, 927.2) = 4.13, *p* < 0.001; Wilk’s Λ = 0.29), as well as in the interaction between profession and labor ward (F(120, 1380.7) = 1.65, *p* < 0.001; Wilk’s Λ = 0.35). Similarly, for the ORC-SWE-S dimensions, there was a statistically significant difference based on profession (F(14, 1016) = 9.26, *p* < 0.001; Wilk’s Λ = 0.79) and labor ward (F(42, 3078) = 8.78, *p* < 0.001; Pillai’s trace = 0.64), as well as their interaction (F(84, 36) = 2.68, *p* < 0.001; Pillai’s trace = 0.41).

The proportion of “do not know” responses for items in the HSOPS 2.0 was below 10%, with the exception of the dimension *Reporting of Patient Safety Events*, where “do not know” responses ranged from 28.5% to 31.9%, and *Hospital Management Support for Patient Safety*, which ranged from 16.4% to 22.0% (Supplementary Material Table S3). Missing data ranged from 4.8% to 6.4% for the HSOPS 2.0 and from 2.0% to 4.7% for the ORC-SWE-S (Supplementary Material, Table S3, S4).

As shown in Table 3, statistically significant differences were observed in how midwives, physicians, and nurse assistants rated patient safety culture and ORC; although all effect sizes were small (η² < 0.05), indicating modest group differences. Overall, all staff, regardless of profession, rated the patient safety culture positively. The dimension with the highest ratings was *Teamwork*, with midwives scoring it significantly higher (4.29 ± 0.56) than physicians (4.13 ± 0.57). Conversely, the dimension with the lowest ratings was *Hospital Management Support for Patient Safety*. Additionally, physicians rated the *Staffing and Work Pace* dimension lower (2.98 ± 0.87) compared to midwives (3.29 ± 0.89) (η² = 0.04).

**Table 3:** Main effect of profession on patient safety culture (HSOPS 2.0) and organizational readiness for change (ORC-SWE-S) assessed by Two-way ANOVA

HSOPS 2.0 dimensions	Midwife *n* = 312	Physician *n* = 153	Nurse Assistant *n* = 180	F-value	*p*-value	Tukey’s HSD^1^	Eta Squared^2^
	Mean (SD)	Mean (SD)	Mean (SD)				
Teamwork	4.29 (0.56)	4.13 (0.57)	4.17 (0.62)	4.28	< 0.001	MW vs. PH (*p* = 0.02)	0.02
Staffing and Work Pace	3.29 (0.89)	2.98 (0.87)	3.14 (0.77)	10.68	< 0.001	MW vs. PH (*p* < 0.01) PH vs. NAs (*p* = 0.01)	0.04
Organizational Learning	3.49 (0.79)	3.69 (0.75)	3.62 (0.75)	3.23	0.05		0.01
Response to Error	3.51 (0.72)	3.53 (0.76)	3.40 (0.54)	1.28	0.28		0.01
Leader Support for Patient Safety	3.85 (0.84)	3.95 (0.71)	3.89 (0.73)	0.54	0.58		< 0.01
Communication about Error	3.75 (0.80)	3.68 (0.86)	3.73 (0.80)	0.79	0.45		< 0.01
Communication Openness	3.64 (0.59)	3.76 (0.52)	3.77 (0.55)	3.27	0.04	MW vs. NAs (*p* = 0.05)	0.01
Reporting Patient Safety Events	3.21 (0.90)	3.23 (0.94)	3.49 (0.94)	4.45	0.01	MW vs. NAs (*p* = 0.03) PH vs. NAs (*p* = 0.02)	0.02
Hospital Management Support for Patient Safety	2.69 (0.83)	2.69 (0.83)	2.99 (0.74)	4.85	0.01	PH vs. NAs (*p* = 0.02) MW vs. NAs (*p* = 0.02)	0.02
Handoffs and Information Exchange	3.72 (0.67)	3.40 (0.76)	3.67 (0.69)	8.28	< 0.001	MW vs. PH (*p* < 0.01) PH vs. NAs (*p* = 0.01)	0.03
ORC-SWE-S dimensions	Mean (SD)	Mean (SD)	Mean (SD)				
Organizational Trust	3.42 (0.58)	3.25 (0.55)	3.64 (0.50)	22.84	< 0.001	MW vs. PH (*p* < 0.01) NAs vs. MW (*p* < 0.01) NAs vs. PH (*p* < 0.01)	0.07
Inadequate Staffing Resources	3.05 (0.98)	3.39 (0.82)	3.17 (0.87)	14.85	< 0.001	PH vs. MW (*p* < 0.01) PH vs. NAs (*p* < 0.01)	0.05
Workplace Satisfaction	4.40 (0.45)	4.19 (0.50)	4.45 (0.46)	13.68	< 0.001	MW vs. PH (*p* < 0.01) NAs vs. PH (*p* < 0.01)	0.05
Role Model	3.96 (0.72)	3.88 (0.68)	3.95 (0.59)	0.47	0.63		< 0.01
Staff and Workplace Challenges	3.17 (0.84)	3.30 (0.77)	3.21 (0.82)	0.88	0.42		< 0.01
Facilities and Equipment	2.92 (0.83)	3.24 (0.79)	3.19 (0.70)	14.73	< 0.001	PH vs. MW (*p* < 0.01) NAs vs. MW (*p* < 0.01)	0.05
External Demand for Change	3.03 (0.81)	2.94 (0.89)	2.94 (0.89)	2.07	0.13		< 0.01

The HSOPS 2.0 and the ORC-SWE-S scales uses a 5-point Likert scale; from 1 (strongly disagree) to 5 (strongly agree). For all HSOPS dimensions higher values indicate stronger agreement

For the ORC-SWE-S dimensions Organizational Trust, Workplace Satisfaction, Role Model, and Facilities and Equipment higher values indicate stronger agreement

For the ORC-SWE-S dimensions Inadequate Staffing resources, Staff and Workplace Challenges, and External Demand for Change higher values indicates stronger disagreement

^1^For Tukey HSD, only tests with p-values < 0.05 are provided

^2^Effect size according to Cohen: small to medium: η²=0.01–0.05, moderate to large: η²=0.06–0.13, large: η²= 0.14

ORC was also rated positively, with the highest scores among staff for the dimension *Workplace Satisfaction*, which was rated highest by nurse assistants (4.45 ± 0.46). The dimension with the highest effect size was the dimension *Organizational Trust* (F = 22.84, *p* < 0.001, η²=0.07), which measures staff confidence in their workplace environment, leadership, and decision-making, including freedom to experiment, autonomy, and organizational adaptability to change (Supplementary Material Table S2). For this dimension the physicians scored lowest (3.25 ± 0.55). Midwives on the other hand scored lowest on the dimension *“Facilities and Equipment”* (2.92, ± 0.83) (Table 3).

When assessing the effect of organization on patient safety culture and ORC, the largest differences were observed in the HSOPS 2.0 dimension *Staffing and Work Pace* (F = 67.98, *p* < 0.001, η² = 0.43) and the ORC-SWE-S dimension *Inadequate Staffing Resources* (F = 65.03, *p* < 0.001, η² = 0.40), both demonstrating substantial effect sizes. The two largest labor wards in the study, sites A and C, with annual birth rates of 4691 and 3885, respectively, reported the lowest ratings on *Staffing and Work Pace* (2.76 ± 0.63 and 2.50 ± 0.60, respectively), as well as on the *Inadequate Staffing Resources* dimension (3.67 ± 0.69 and 3.93 ± 0.64, respectively) (Table 4).

**Table 4:** Main effect of labor ward (organization) on patient safety culture (HSOPS 2.0) and Organizational Readiness for Change (ORC-SWE-S) assessed by Two-way ANOVA

HSOPS 2.0 dimensions	Labor ward A *n* = 112		Labor ward B *n* = 108		Labor ward C *n* = 120	Labor ward D *n* = 71	Labor ward E *n* = 75	Labor ward F *n* = 95	Labor ward G *n* = 67	F-value	*p*-value	Tukey’s HSD^1,2^	Eta Squa-red^3^
	Mean (SD)		Mean (SD)		Mean (SD)	Mean (SD)	Mean (SD)	Mean (SD)	Mean (SD)				
Teamwork	4.04 (0.55)		4.22 (0.56)		4.26 (0.61)	3.92 (0.63)	4.56 (0.38)	4.48 (0.43)	4.05 (0.61)	12.20	0.01	A vs. E (*p* < 0.01) B vs. D (*p* = 0.03) B vs. E (*p* < 0.01) C vs. D (*p* < 0.01) C vs. E (*p* = 0.03) F vs. A (*p* < 0.01) F vs. B (*p* = 0.01) F vs. D (*p* < 0.01) F vs. G (*p* < 0.01) D vs. E (*p* < 0.01) G vs. E (*p* < 0.01)	0.12
Staffing and Work Pace	2.76 (0.63)		3.34 (0.61)		2.50 (0.60)	3.58 (0.71)	3.44 (0.72)	4.24 (0.52)	2.63 (0.67)	67.98	< 0.001	A vs. B (*p* < 0.01) A vs. D (*p* < 0.01) A vs. E (*p* < 0.01) C vs. A (*p* < 0.01) C vs. B (*p* < 0.01) C vs. E (*p* < 0.01) C vs. D (*p* < 0.01) F vs. A (*p* < 0.01) F vs. B (*p* < 0.01) F vs. C (*p* < 0.01) F vs. D (*p* < 0.01) F vs. E (*p* < 0.01) F vs. G (*p* < 0.01) G vs. B (*p* < 0.01) G vs. D (*p* < 0.01) G vs. E (*p* < 0.01)	0.43
Organizational Learning	3.23 (0.72)		3.51 (0.72)		3.36 (0.81)	3.70 (0.72)	3.94 (0.60)	4.03 (0.68)	3.37 (0.71)	8.52	< 0.001	A vs. E (*p* = 0.02) C vs. E (*p* = 0.03) F vs. A (*p* < 0.01) F vs. B (*p* < 0.01) F vs. C (*p* < 0.01) F vs. G (*p* < 0.01) G vs. E (*p* = 0.02)	0.10
Response to Error	3.22 (0.67)		3.37 (0.66)		3.41 (0.69)	3.63 (0.67)	3.73 (0.62)	3.74 (0.64)	3.46 (0.65)	3.58	0.002	B vs. E (*p* = 0.03) F vs. B (*p* < 0.01)	0.05
Leader Support for Patient Safety	3.58 (0.77)		4.12 (0.70)		3.45 (0.78)	4.36 (0.60)	4.21 (0.57)	3.94 (0.71)	3.83 (0.86)	10.16	< 0.001	A vs. B (*p* < 0.01) A vs. D (*p* < 0.01) A vs. E (*p* = 0.01) C vs. B (*p* < 0.01) C vs. D (*p* < 0.01) C vs. E (*p* < 0.01) F vs. C (*p* < 0.01) G vs. D (*p* = 0.04)	0.11
Communication about Error	3.47 (0.83)		3.54 (0.84)		3.58 (0.69)	3.72 (0.81)	3.97 (0.63)	4.40 (0.56)	3.45 (0.87)	11.81	< 0.001	A vs. E (*p* = 0.01) B vs. E (*p* = 0.04) F vs. A (*p* < 0.01) F vs. B (*p* < 0.01) F vs. C (*p* < 0.01) F vs. D (*p* < 0.01) F vs. E (*p* = 0.02) F vs. G (*p* < 0.01) G vs. E (*p* = 0.03)	0.12
Communication Openness	3.61 (0.62)		3.61 (0.54)		3.58 (0.56)	3.79 (0.53)	3.92 (0.45)	3.97 (0.50)	3.56 (0.57)	5.45	0.001	B vs. E (*p* = 0.04) C vs. E (*p* = 0.03) F vs. A (*p* = 0.01) F vs. B (*p* < 0.01) F vs. C (*p* < 0.01) F vs. G (*p* < 0.01) G vs. E (*p* = 0.04)	0.07
Reporting Patient Safety Events	3.00 (0.80)		3.15 (1.05)		3.10 (0.89)	3.40 (0.96)	3.50 (0.94)	3.61 (0.85)	3.52 (0.85)	3.04	0.007	A vs. G (*p* = 0.05) F vs. A (*p* = 0.03)	0.05
Hospital Management Support for Patient Safety	2.44 (0.85)		2.88 (0.66)		2.57 (0.86)	2.86 (0.86)	3.03 (0.72)	3.17 (0.73)	2.69 (0.68)	5.78	< 0.001	A vs. B (*p* = 0.01) A vs. E (*p* = 0.03) F vs. A (*p* < 0.01) F vs. C (*p* < 0.01)	0.08
Hand-offs and Information Exchange	3.54 (0.65)		3.75 (0.67)		3.45 (0.76)	3.92 (0.66)	3.72 (0.57)	3.75 (0.78)	3.40 (0.65)	4.67	< 0.001	A vs. D (*p* = 0.04) C vs. B (*p* = 0.03) C vs. D (*p* < 0.01) G vs. D (*p* < 0.01)	0.05
ORC-SWE-S dimensions mean (sd)	*n* = 112		*n* = 108		*n* = 120	*n* = 71	*n* = 72	*n* = 95	*n* = 67				
Organizational Trust	3.26 (0.57)		3.50 (0.55)		3.30 (0.53)	3.52 (0.58)	3.77 (0.51)	3.55 (0.51)	3.36 (0.62)	6.10	< 0.001	A vs. E (*p* < 0.01) C vs. E (*p* < 0.01) F vs. A (*p* < 0.01) F vs. C (*p* = 0.02) G vs. E (*p* = 0.01)	0.06
Inadequate Staffing Resources	3.67 (0.69)		3.27 (0.69)		3.93 (0.64)	2.47 (0.73)	2.71 (0.78)	2.19 (0.61)	3.45 (0.68)	65.03	< 0.001	A vs. B (*p* < 0.01) A vs. D (*p* < 0.01) A vs. E (*p* < 0.01) B vs. D (*p* < 0.01) B vs. E (*p* < 0.01) C vs. B (*p* < 0.01) C vs. D (*p* < 0.01) C vs. E (*p* < 0.01) C vs. G (*p* < 0.01) F vs. A (*p* < 0.01) F vs. B (*p* < 0.01) F vs. C (*p* < 0.01) F vs. E (*p* < 0.01) F vs. G (*p* < 0.01) G vs. D (*p* < 0.01) G vs. E (*p* < 0.01)	0.40
Workplace Satisfaction	4.37 (0.50)		4.38 (0.52)		4.35 (0.44)	4.42 (0.43)	4.34 (0.47)	4.44 (0.44)	4.16 (0.49)	2.78	0.01	F vs. G (*p* < 0.01) G vs. D (*p* = 0.04)	0.03
Role Model	4.01 (0.64)		3.94 (0.71)		3.99 (0.65)	3.92 (0.64)	4.34 (0.47)	3.90 (0.72)	3.76 (0.70)	0.86	0.52		0.01
Staff and Workplace Challenge	3.39 (0.78)		3.06 (0.78)		3.23 (0.79)	3.28 (0.78)	2.85 (0.78)	2.95 (0.81)	3.78 (0.68)	9.40	< 0.001	A vs. E (*p* = 0.01) A vs. G (*p* = 0.02) C vs. G (*p* < 0.01) F vs. A (*p* < 0.01) F vs. G (*p* < 0.01) G vs. B (*p* < 0.01) G vs. D (*p* = 0.02) G vs. E (*p* < 0.01)	0.09
Facilities and Equipment	2.79 (0.87)		2.91 (0.83)		2.96 (0.70)	2.88 (0.69)	2.85 (0.78)	3.54 (0.68)	2.95 (0.66)	14.43	< 0.001	A vs. E (*p* < 0.01) B vs. E (*p* < 0.01) C vs. E (*p* < 0.01) D vs. HA (*p* < 0.01) F vs. A (*p* < 0.01) F vs. B (*p* < 0.01) F vs. C (*p* < 0.01) F vs. D (*p* < 0.01) F vs. G (*p* < 0.01) G vs. E (*p* < 0.01)	0.13
External Demand for Change	3.04 (0.82)		3.04 (0.79)		3.31 (0.89)	2.78 (0.87)	2.64 (0.90)	2.75 (0.84)	3.15 (0.60)	8.68	< 0.001	A vs. E (*p* = 0.03) B vs. E (*p* = 0.01) C vs. D (*p* < 0.01) C vs. E (*p* < 0.01) F vs. B (*p* = 0.05) F vs. C (*p* < 0.01) F vs. G (*p* = 0.01) G vs. E (*p* < 0.01)	0.08

The HSOPS 2.0 and the ORC-SWE-S scales uses a 5-point Likert scale; from 1 (strongly disagree) to 5 (strongly agree)

For all HSOPS dimensions higher values indicate stronger agreement

For the ORC-SWE-S dimensions Organizational trust, Workplace Satisfaction, Role Model, and Facilities and Equipment higher values indicate stronger agreement

For the ORC-SWE-S dimensions Inadequate staffing resources, Staff and workplace challenges, and External demand for change higher values indicates stronger disagreement

^1^For Tukey HSD, only tests with p-values < 0.05 are provided

^2^Abbreviations for the labor wards: A: Region Southern Labor ward A, B: Region Southern Labor ward B, C: Region Central Labor ward C, D: Region West-Central Labor ward D, E: Region Southwestern Labor ward E, F: Region Southwestern Labor ward F, G: Region North-Central Labor ward G

^3^Effect size according to Cohen: small to medium: η²=0.01–0.05, moderate to large: η² 0.06–0.13, large: η²= 0.14

In several of the areas related to both patient safety culture and ORC, the Tukey HSD post-hoc test indicated that the Labor Ward F differed significantly from the others, demonstrating more positive attitudes in the patient safety dimensions *Staffing and Work Pace* and *Communication about Error*, as well as the ORC-SWE-S dimensions *Organizational Learning*, *Inadequate Staffing Resources*, and *Facilities and Equipment*. Labor Ward F also had the best results, with the lowest proportion of infants born with Apgar scores < 7 at 5 min and the lowest rate of severe perineal trauma (Table 1). The two labor wards, E and F, located within the same region and overseen by the same management board, reported the most positive attitudes on the *Teamwork* dimension, with mean scores of 4.48 ± 0.43 and 4.56 ± 0.38, respectively (Table 4).

A significant interaction effect between profession and labor ward was identified in six of the dimensions related to patient safety culture and five dimensions of the ORC-SWE-S, indicating that perceptions were influenced by both profession and workplace. The most substantial effects were observed in the HSOPS 2.0 dimension *Staffing and Work Pace* (F = 5.70, *p* < 0.001, η² = 0.11) and the ORC-SWE-S dimension *Inadequate Staffing Resources* (F = 7.97, *p* < 0.001, η² = 0.14). (Table 5). The results of the two-way ANOVA and interaction effects are further visualized in the supplementary material (Supplementary Material, Figure S1, S2, S3).

**Table 5:** Interaction between profession and organization (labor ward) for the dimensions measuring hospital survey on patient safety (HSOPS 2.0) and organizational readiness for change (ORC-SWE-S)

	F-value	*p*-value		Eta Squared^1^	
**HSOPS 2.0**					
Teamwork	2.10	0.02		0.04	
Staffing and Work Pace	5.70	< 0.001		0.11	
Organizational Learning	3.43	< 0.001		0.08	
Response to Error	2.65	0.01		0.07	
Leader Support for Patient Safety	3.69	< 0.001		0.08	
Communication about Error	1.90	0.03		0.04	
Communication about Openness	1.19	0.29		0.03	
Reporting Patient Safety Events	1.68	0.07		0.05	
Hospital Management Support for Patient Safety	1.09	0.36		0.03	
Handoffs and Information Exchange	1.20	0.28		0.03	
ORC-SWE-S					
Organizational Trust	3.26	< 0.001		0.06	
Staffing Resources	7.97	< 0.001		0.14	
Workplace Satisfaction	1.28	< 0.001		0.03	
Role Model	0.98	0.47		0.02	
Staff and Workplace Challenges	1.74	0.06		0.03	
Facilities and Equipment	2.32	< 0.01		0.05	
External Demand for Change	2.95	< 0.001		0.06	

^1^Effect size according to Cohen: small η²=0.01, moderate η² 0.06, large η²= 0.14

For the single items in HSOPS 2.0, there were statistically significant differences for *Number of Patient Safety Events Reported During the Past 12 Months* when comparisons were made both by profession (*p* < 0.001) and by labor ward (*p* = 0.03), whereas the *Overall Rating of Patient Safety on the Labor Ward* was only statistically significant for comparisons between labor wards (*p* < 0.001). For example, 36.5% of midwives reported not having reported any patient safety events during the past year, compared to 85.9% of nurse assistants and 46.2% of physicians. In Labor Ward B, 71.3% of the staff stated that they had not reported any patient safety events during the past year, compared to 38.5% in Labor Ward G.

Among the dimensions of the ORC-SWE-S, *Organizational Trust* was positively correlated with the HSPOS dimensions *Organizational Learning* (*r* = 0.55), *Leader Support for Patient Safety* (*r* = 0.56), *Communication about Error* (*r* = 0.53), and *Communication Openness* (*r* = 0.53). Negative correlations were found between the ORC-SWE-S dimension *Inadequate Staffing Resources* and the HSOPS 2.0 dimensions *Staffing and Work Pace* (*r* = -0.79) and *Organizational Learning* (*r* = -0.59), as well as between *Staff and Workplace Challenges* and *Teamwork* (*r* = -0.53) (Table 6).

**Table 6:** Correlation between the hospital survey on patient safety (HSOPS 2.0) and organizational readiness for change (ORC-SWE-S) dimensions

HSOPS 2.0	Organizational Trust	Staffing Resources	Workplace Satisfaction	Role Model	Staff and Workplace Challenges	Facilities and Equipment	External Demand for Change
Team-work	*N* = 547 0.38	*N* = 549 -0.30	*N* = 533 0.30	*N* = 554 0.19	*N* = 550 -0.53	*N* = 551 0.26	*N* = 550 -0.16
Staffing and Work Pace	*N* = 546 0.39	*N* = 549 -0.79	*N* = 531 0.17	*N* = 553 0.09	*N* = 546 -0.32	*N* = 550 0.34	*N* = 549 -0.23
Org. Learning	*N* = 486 0.55	*N* = 487 -0.59	*N* = 471 0.19	*N* = 489 0.16	*N* = 485 -0.26	*N* = 488 0.39	*N* = 487 -0.12
Response to Error	*N* = 425 0.44	*N* = 428 -0.42	*N* = 410 0.23	*N* = 430 0.22	*N* = 430 -0.33	*N* = 428 0.26	*N* = 428 -0.08
Leader Support for Patient Safety	*N* = 511 0.56	*N* = 515 -0.48	*N* = 499 0.31	*N* = 516 0.25	*N* = 512 -0.24	*N* = 516 0.29	*N* = 511 -0.06
Communi-cation about Error	*N* = 525 0.53	*N* = 531 -0.48	*N* = 513 0.26	*N* = 532 0.17	*N* = 530 -0.37	*N* = 531 0.34	*N* = 530 -0.08
Communi-cation about Openness	*N* = 450 0.53	*N* = 455 -0.49	*N* = 439 0.24	*N* = 457 0.24	*N* = 456 -0.34	*N* = 457 0.36	*N* = 453 -0.14
Reporting Patient Safety Events	*N* = 377 0.42	*N* = 383 -0.30	*N* = 366 0.25	*N* = 383 0.23	*N* = 382 -0.29	*N* = 382 0.10	*N* = 379 -0.03
Hospital Manage-ment Support for Patient Safety	*N* = 423 0.38	*N* = 428 -0.40	*N* = 411 0.02	*N* = 428 0.05	*N* = 427 -0.20	*N* = 424 0.35	*N* = 426 0.01
Hand-offs and Infor-mation Exchange	*N* = 534 0.21	*N* = 536 -0.34	*N* = 522 0.21	*N* = 541 0.16	*N* = 536 -0.22	*N* = 538 0.15	*N* = 538 -0.23

Note: Strength of correlation interpreted according to Cohen: Small correlation: *r* = 0.10 to < 0.30, Medium correlation: *r* = 0.30 to < 0.50, Large correlation: r = > 0.50

## Discussion

Overall, in this study, patient safety culture and ORC were rated positively, with perceptions influenced by both profession and organization. An interaction effect between profession and organization was found for six of the ten patient safety culture dimensions and five of the seven ORC dimensions, indicating that profession and organization together shaped the perceptions. Organization however, had a greater impact than profession. Among the patient safety culture dimensions, *Teamwork* received the highest scores, while *Staffing and Workpace* (HSOPS 2.0) and *Inadequate Staffing Resources* (ORC-SWE-S) showed the most significant differences between labor wards.

The finding that profession and organization influence patient safety culture aligns with previous research [38–40]. The results highlight the importance of understanding the context in which the implementation will be initiated. Factors related to the *Inner Setting* in CFIR, including structural characteristics, resources, adequate staffing, and implementation climate (i.e., readiness for change) [29], may shape patient safety culture by enabling its development when these conditions are sufficient, or hindering it when they are lacking [13, 41]. This is illustrated by Labor Wards E and F, located within the same region and under shared management, reporting the most positive perceptions of patient safety culture and ORC.

Dimensions that potentially have a significant negative impact on implementation and patient safe culture and therefore warrant particular attention include *Staffing and Work Pace* (HSOPS 2.0), and *Inadequate Staffing Resources* dimension in ORC-SWE-S, both strongly correlated in this study. These dimensions reflect a high workload, which is a well-documented barrier to the implementation of new practices, as it constrains time, attention, and staff capacity for change [24, 42]. Labor wards A and C reported the lowest scores on both dimensions; both are large units characterized by a high annual birth volume and a correspondingly large workforce. High workload and shortage of staff are known barriers for change management activities and educational efforts, while stress and high workload may adversely affect engagement among staff for adopting new practices or adhering to new routines [42, 43]. Previous literature suggests that low scores across ORC dimensions indicate limited readiness and that implementation efforts should ideally be postponed until key challenges are addressed [44]. However, given the increasing complexity of healthcare and the constraints commonly present in clinical settings such as maternity care, delaying implementation or allocating additional resources may not always be feasible. The purpose of using the ORC-SWE-S as a baseline measure prior to the implementation of the TeamBirth care process was to identify which dimensions should be considered when introducing this care process. At the same time, the results may also be valuable for the implementation of other interventions that require behavioral change among staff. The results can thereby be used to provide managers and staff responsible for implementation with an increased understanding of which areas of the *Inner Setting* [24] should be addressed, and to inform the selection of targeted implementation strategies. For example, profession appeared less influential than workplace, an important consideration in the choice of implementation strategies [26]. Having staff score high on *Organizational Trust* may also be beneficial, as this dimension has been identified as a facilitator for implementation [42, 43].

Consistent with findings from other studies, Teamwork received the highest ratings among the patient safety culture dimensions [40, 45]. Interestingly this is an area in intrapartum care where deficiencies are frequently identified and are known to contribute to adverse outcomes for women and their infants [46, 47]. However, irrespective of how respondents answer teamwork items related to patient safety, a high rating does not necessarily reflect the quality of teamwork in all situations. Staff may feel that they work well together in day-to-day practice yet fail to recognize latent risks embedded in hierarchical structures, role clarity, or communication pathways [48, 49]. Additionally, midwives rated teamwork higher than physicians. Reviews of interprofessional collaboration reveal conflicting findings, with some studies indicating that physicians rate teamwork more positively, while others suggest the opposite [38, 50]. The reasons for the conflicting results may be due to context, although hierarchy has also been reported as one reason for differing views on whether team collaboration is good or not [51]. Different philosophies of care are known to affect and challenge collaboration between midwives and physicians [52] but may be less pronounced when teamwork is evaluated in the context of patient safety. Nevertheless, care philosophies reflect underlying values and norms inherent in the concept of culture and are therefore also relevant to patient safety culture [13]. In Sweden, maternity care has traditionally been, and largely remains, structured with midwives and nurse assistants belonging to one part of the organization, while obstetricians and residents belong to another. These groups often work different hours, attend separate meetings, and engage in distinct training activities, reflecting structural characteristics and communication patterns. This organizational division has been identified as a barrier for teamwork, with both obstetricians and midwives describing their professional identities as shaped by separate domains, differing role perceptions, structural divisions, and existing hierarchies [48]. This underscores the importance of creating shared forums for discussion that facilitate communication and mutual understanding, thereby supporting interprofessional collaboration and alignment in everyday clinical practice. Such forums may help address role conflicts previously reported [53], which, if unaddressed, may compromise patient safety and the implementation of new care interventions such as TeamBirth. As all professional groups scored high on the ORC-SWE-S dimensions *Workplace Satisfaction* and *Role Model*, this indicates satisfaction with both their assigned tasks and their role within the workplace. These dimensions can further be connected to enabling factors for patient safety, as they relate to group belonging, appreciation at work, and acting as a role model, thereby contributing to the shaping of norms and values related to patient safety culture at the group level [13].

Finally, we found several of the dimensions in HSOPS 2.0 and ORC-SWE-S were strongly correlated, supporting the idea of the connection between patient safety culture and organizational culture related to change. For example, the observed correlation between *Organizational Trust* and *Organizational Learning* suggests that labor wards in which staff have autonomy in decision-making, feel encouraged, and perceive that their ideas are valued are also organizations that promote reflection, learning, and continuous improvement to prevent adverse events and improve outcomes.

### Strengths and limitations

The strengths of this study include the inclusion of seven labor wards of varying sizes across five regions, representing both rural and urban areas, which contributes to the generalizability of the findings. The high response rate among staff further supports the representativeness of the sample and thereby enhances the reliability of the study findings. Additionally, the use of validated scales strengthens the validity of the measurement of patient safety culture and ORC.

However, several limitations should be acknowledged. First, the use of a cross-sectional design limits the ability to draw any causal inferences. Second, reliance on self-reported data may not fully reflect actual behaviors related to patient safety or organizational practices since it may introduce bias, for example recall bias or social desirability bias. Within the HSOPS 2.0 instrument, an item potentially susceptible to recall bias is “*the number of patient safety events reported by an individual over the past year.”* However, in the present study, this item was not given substantial weight, as we considered it more informative from a quality improvement perspective than for analytical comparisons. With regard to social desirability bias, this response tendency is typically associated with sensitive topics or socially normative expectations at the individual level [54]. It can be discussed to what extent this applies to instruments such as HSOPS 2.0 and ORC-SWE-S, which are designed to capture perceptions of climate and organizational attitudes rather than individual behavior [14, 55]. Nevertheless, certain dimensions of patient safety culture may be more influenced by normative expectations, particularly those related to teamwork and communication [56]. Notably, the HSOPS 2.0 teamwork dimension received the highest ratings across professions and labor wards, suggesting that this aspect of safety culture may warrant further investigation using complementary perspectives and alternative methodological approaches. Incorporating interviews with key personnel or conducting focus group discussions at the study sites could have provided a deeper understanding of the underlying contextual factors influencing the findings [57].

Third, the analyses did not adjust for potential confounders such as age, work experience, or shift work, which may influence perceptions of safety culture and organizational readiness for change. The available data were considered insufficient to support adjusted analyses without risking model instability, in particular with regard to organization, i.e., labor ward. Instead, the results were interpreted using post hoc comparisons and effect sizes, while acknowledging the limitations associated with unadjusted analyses.

Fourth, the use of a scale that is currently undergoing validation should be considered a limitation. The original ORC-S consists of 115 items [34] and includes content considered outdated; therefore, a shortened and updated Swedish version was developed to allow concurrent baseline assessment of organizational readiness for change and patient safety culture. As the psychometric validation of this revised instrument has not yet been published, uncertainty remains regarding the construct validity of findings related to organizational readiness for change.

Finally, although the study included multiple labor wards, the findings may not be fully generalizable to other maternity care settings, particularly outside Sweden. Differences in contextual conditions related to implementation and readiness for change need to be considered when interpreting the findings.

## Conclusion

Both profession and organization influenced how staff rated patient safety culture and ORC; however, organization had a greater impact. Among the patient safety culture dimensions, *Teamwork* received the highest ratings among the patient safety culture dimensions, while *Inadequate Staffing Resources*, as well as *Staffing and Work Pace*, showed the greatest variation between labor wards. Dimensions related to workload and staffing emerged as key barriers to implementation. Assessing patient safety culture and organizational readiness for change may support the tailoring of implementation strategies for complex interventions such as TeamBirth.

## Supplementary Information

Below is the link to the electronic supplementary material.

Supplementary Material 1

## Data Availability

The datasets generated and/or analyzed during the current study are not publicly available owing to the lack of ethical approval for public dissemination, but they are available from the corresponding author upon reasonable request.

## Abbreviations

ORC: Organizational Readiness for Change
HSOPS 2.0: Hospital Survey On Patient Safety
AHRQ: Agency for Healthcare Research and Quality
IVO: Swedish Health and Social Care Inspectorate
CFIR: Consolidated Framework for Implementation Research
