# Supplementary material for: Ready for change? The effect of profession and organization on patient safety culture and organizational readiness for change – a cross-sectional study
Source: BMC Health Serv Res. 2026 Feb 18;26:307. doi: 10.1186/s12913-026-14187-w (PMC12937508; doi:10.1186/s12913-026-14187-w)
Supplement: Supplementary file 1 — Supplementary Material 1 [file 12913_2026_14187_MOESM1_ESM.docx]

## SuPPLEMENTARY MATERIAL


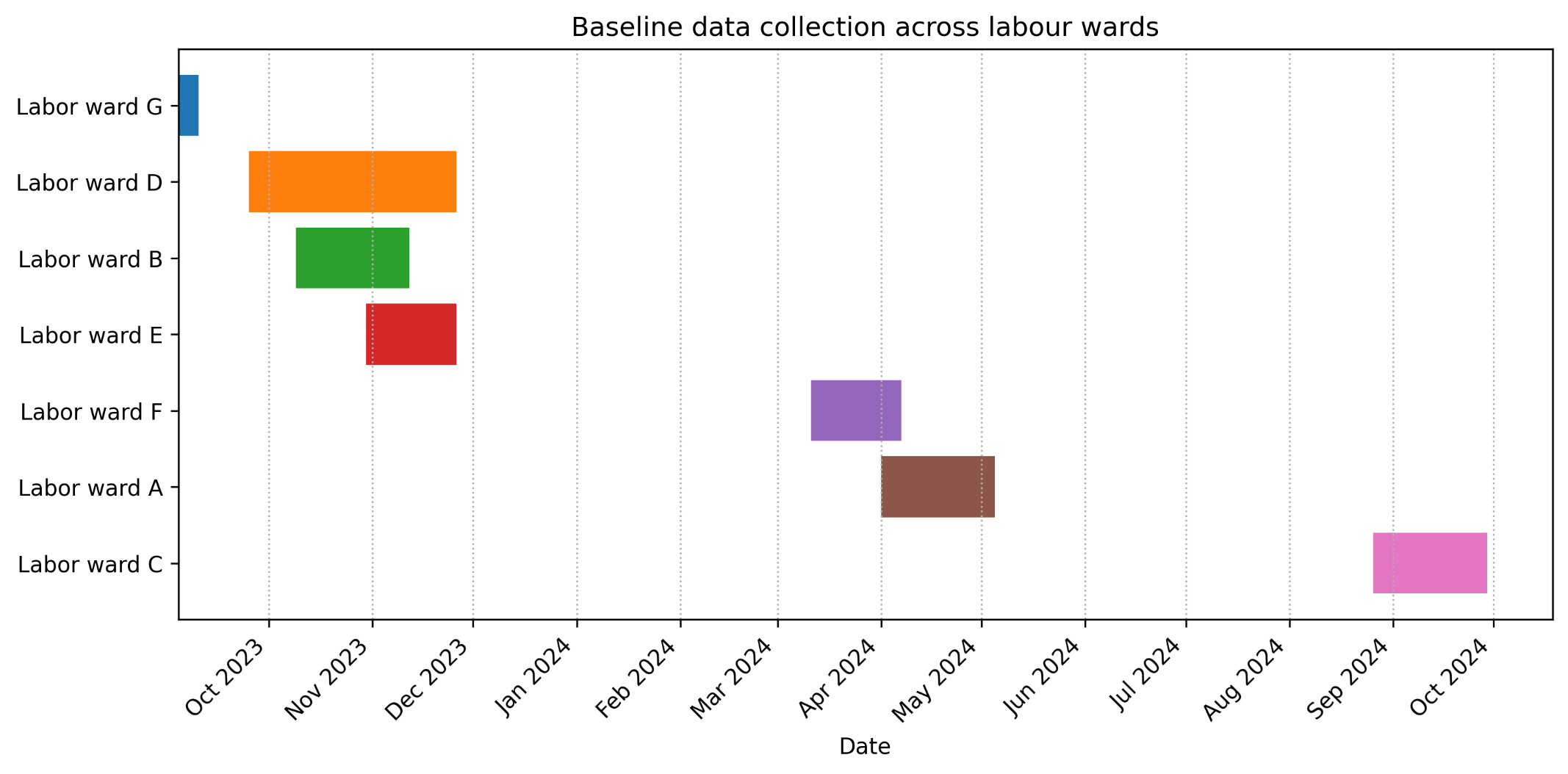


**Figure S1.** Overview of baseline data collection periods prior to implementation across participating labor wards

The labor wards joined the project at different time points depending on what worked for each respective organization. Data collection continued until a response rate of approximately 75% was achieved, which was achieved between 4 to 9 weeks. At labor ward G, data collection was carried out during a training initiative for all staff over the course of one week. At the other sites, data collection took place over a longer period of time

|  | |  |  |
| --- | --- | --- | --- |
| **Table S1. Dimensions and items in the Hospital Survey On Patient Safety Culture (HSOPS 2.0)** | | |  |
| **Dimension** | | | Chronbach’s alpha |
| **1** | **Teamwork** | | 0.76 |
|  | In this unit, we work together as an effective team. | |  |
|  | During busy times, staff in this unit help each other | |  |
|  | There is a problem with disrespectful behavior by those working in this unit (R) | |  |
| **2** | **Staffing and Work Pace** | | 0.67 |
|  | In this unit, we have enough staff to handle the workload | |  |
|  | Staff in this unit work longer hours than is best for patient care | |  |
|  | This unit relies too much on temporary staff (R) | |  |
|  | The work pace in this unit is so rushed that it negatively affects patient safety (R) | |  |
| **3** | **Organizational Learning – Continuous Improvement** | | 0.76 |
|  | This unit regularly reviews work processes to determine if changes are needed to improve patient safety. | |  |
|  | In this unit, changes to improve patient safety are evaluated to see how well they worked | |  |
|  | This unit lets the same patient safety problems keep happening (R) | |  |
| **4** | **Response to Error** | | 0.83 |
|  | In this unit, staff feel like their mistakes are held against them (R) | |  |
|  | When an event is reported in this unit, it feels like the person is being written up, not the problem (R) | |  |
|  | When staff make errors, this unit focuses on learning rather than blaming individuals | |  |
|  | In this unit, there is a lack of support for staff involved in patient safety errors (R) | |  |
| **5** | **Leader Support for Patient Safety** | | 0.77 |
|  | My supervisor, manager, or clinical leader seriously considers staff suggestions for improving patient safety | |  |
|  | My supervisor, manager, or clinical leader wants us to work faster during busy times, even if it means taking shortcuts. | |  |
|  | My supervisor, manager, or clinical leader takes action to address patient safety concerns that are brought to their attention | |  |
| **6** | **Communication About Error** | | 0.89 |
|  | We are informed about errors that happen in this unit | |  |
|  | When errors happen in this unit, we discuss ways to prevent them from happening again | |  |
|  | In this unit, we are informed about changes that are made based on event reports | |  |
| **7** | **Communication Openness** | | 0.83 |
|  | In this unit, staff speak up if they see something that may negatively affect patient care | |  |
|  | When staff in this unit see someone with more authority doing something unsafe for patients, they speak up. | |  |
|  | When staff in this unit speak up, those with more authority are open to their patient safety concerns. | |  |
|  | In this unit, staff are afraid to ask questions when something does not seem right. (R) | |  |
| **8** | **Reporting Patient Safety Events** | | 0.75 |
|  | When a mistake is caught and corrected before reaching the patient, how often is this reported? | |  |
|  | When a mistake reaches the patient and could have harmed the patient, but did not, how often is this reported? | |  |
| **9** | **Hospital Management Support for Patient Safety** | | 0.77 |
|  | The actions of hospital management show that patient safety is a top priority | |  |
|  | Hospital management provides adequate resources to improve patient safety | |  |
|  | Hospital management seems interested in patient safety only after an adverse event | |  |
| **10** | **Handoffs and Information Exchange** | | 0.72 |
|  | When transferring patients from one unit to another, important information is often left out (R) | |  |
|  | During shift changes, important patient care information is often left out (R) | |  |
|  | During shift changes, there is adequate time to exchange all key patient care information | |  |

**Single items**

In the past 12 months, how many patient safety events have you reported?

□ None □ 1-2 □ 3-5 □ 6-10 □ 11 or more

How would you rate your unit/work area on patient safety?

□ Poor □ Fair □ Good □ Very good □ Excellent

HSOPS 2.0 Items are rated on a 5-point Likert scale; from 1 (strongly disagree) to 5 (strongly agree) and a response option for “does not apply/don’t know. The responses “does not apply/don’t know were treated as missing.
R= reversed when scores are calculated

| Table S2. Dimensions and items – Organizational Readiness for Change scale* (ORC-SWE-S) | |  |
| --- | --- | --- |
| **Dimension** | | Chronbach’s alpha |
| **1** | **Organizational trust** | 0.77 |
|  | Staff have the autonomy to try out different ideas or ways of working |  |
|  | Managers are receptive to ideas and suggestions from the staff |  |
|  | Staff have significant autonomy to make independent decisions at work |  |
|  | Management makes well-informed decisions |  |
|  | Our organization has clear and well-defined goals |  |
|  | Management has a clear plan for how we are to achieve our goals |  |
|  | Changing routines to match new requirements is straightforward |  |
| **2** | **Inadequate Staffing Resources** | 0.84 |
|  | My workplace has enough staff to meet the patients' needs (R) |  |
|  | Staff at my workplace usually have enough time to do their work (R) |  |
|  | I have too much workload to do a good job |  |
|  | Staff often show symptoms of stress or burn out |  |
|  | My workplace has problems with high staff turnover |  |
| **3** | **Workplace Satisfaction** | 0.73 |
|  | I highly value my contribution at work |  |
|  | I enjoy my work |  |
|  | I have a positive impact on my colleagues |  |
|  | Learning and using new procedures and routines is easy for me |  |
|  | I feel appreciated for the work I do |  |
| **4** | **Role Model** | 0.78 |
|  | Other staff often ask for my advice |  |
|  | I am viewed as a leader by the staff I work with |  |
|  | I am considered an experienced source of advice in my field |  |
|  | I frequently share my knowledge and ideas with others |  |
| **5** | **Staff and Workplace Challenges** | 0.79 |
|  | My organization needs guidance in improving relations among staff |  |
|  | My organization needs guidance in improving communications among staff |  |
|  | There is too much conflict between different professions |  |
|  | My organization needs guidance in evaluating staff performance |  |
| **6** | **Facilities & Equipment** | 0.75 |
|  | Computer and IT problems are usually repaired promptly at my workplace |  |
|  | Clinical equipment at my workplace is adequate (for example CTG monitors, ultrasound or other medical-technical equipment) |  |
|  | Problems with clinical equipment are usually repaired promptly at my workplace |  |
|  | My work facilities are adequate |  |
|  | The staff are satisfied with our computer systems |  |
| **7** | **External Demand for Change** | 0.77 |
|  | Current pressure to make changes comes from politicians |  |
|  | Current pressure to make changes comes from my manager/managers |  |
|  | Current pressure to make changes comes from hospital management |  |

*Shortened and modified version of the ORC-SWE scale
Items are rated on a 5-point Likert scale; from 1 (strongly disagree) to 5 (strongly agree).

**Table S3. Overview of the background questions included in the survey**

1. I work as:

□ Nurse assistant □ Midwife □ Obstetrician □ Gynecologist □ Resident

1. I am:
   □ <26 years old □ 26–35 years old □ 36–45 years old □ 46–55 years old □ >55 years old
2. I identify as:
   □ Woman □ Man □ Other
3. I work at the following maternity ward:
   □ Malmö □ Lund □ Uppsala □ Karlstad □ Halmstad □ Varberg □ Östersund
4. I work:
   **** Day/evening **** Rotational shifts **** Only night shifts
   **** Other, specify: ______________________________
5. What type of employment do you have?
   **** Permanent **** Temporary **** Hourly Employment **** Other, specify: ____________
6. Approximately how much do you work at this labour ward?

   Please specify in %: _____________
7. How long have you worked in maternity care?

   **** Less than 6 months **** 6–11 months **** 1–2 years **** 3–5 years **** 6–10 years **** 11–20 years **** 20 years or more
8. Typically, how many hours per week do you work at this hospital?
   **** Less than 30 hours per week **** 30 to 40 hours per week **** More than 40 hours per week

1. I have been employed at the clinic (answer based on your current profession)

   **** Less than 6 months **** 6–11 months **** 1–5 years **** 6–10 years **** 11 years or more
2. I have been employed at the hospital (answer based on your current profession)

   **** Less than 6 months **** 6–11 months **** 1–5 years **** 6–10 years **** 11 years or more
3. In your professional role, do you typically have direct contact with patients?
   **** Yes, I usually have direct contact with patients **** No, I usually do NOT have direct contact with patients

**Table S4.** Missing data and “does not apply/do not know” response rates for the Hospital Survey On Patient Safety Culture (HSOPS 2.0)

|  | N=645 | Does not apply/do not know | Missing data |
| --- | --- | --- | --- |
| 1 | In this unit, we work together as an effective team | 8 (1.2%) | 32 (5.0%) |
| 2 | In this unit, we have enough staff to handle the workload | 6 (0.9%) | 32 (5.0%) |
| 3 | Staff in this unit work longer hours than is best for patient care | 15 (2.3%) | 33 (5.1%) |
| 4 | This unit regularly reviews work processes to determine if changes are needed to improve patient safety | 36 (5.6%) | 31 (4.8%) |
| 5 | This unit relies too much on temporary staff | 31 (4.8%) | 34 (5.3%) |
| 6 | In this unit, staff feel like their mistakes are held against them^1^ | 61 (9.5%) | 31 (4.8%) |
| 7 | When an event is reported in this unit, it feels like the person is being written up, not the problem | 51 (7.9%) | 33 (5.1%) |
| 8 | During busy times, staff in this unit help each other | 33 (5.1%) | 0 (0.0%) |
| 9 | There is a problem with disrespectful behavior by those working in this unit | 29 (4.5%) | 31 (4.8%) |
| 10 | The work pace in this unit is so rushed that it negatively affects patient safety | 7 (1.1%) | 33 (5.1%) |
| 11 | In this unit, changes to improve patient safety are evaluated to see how well they worked | 51 (7.9%) | 35 (5.4%) |
| 12 | In this unit, there is a lack of support for staff involved in patient safety errors | 139 (21.6%) | 32 (5.0%) |
| 13 | This unit lets the same patient safety problems keep happening | 75 (11.6%) | 34 (5.3%) |
| 14 | My supervisor, manager, or clinical leader seriously considers staff suggestions for improving patient safety | 49 (7.6%) | 36 (5.6%) |
| 15 | My supervisor, manager, or clinical leader wants us to work faster during busy times, even if it means taking shortcuts | 40 (6.2%) | 33 (5.1%) |
| 16 | My supervisor, manager, or clinical leader takes action to address patient safety concerns that are brought to their attention | 50 (7.8%) | 35 (5.4%) |
| 17 | We are informed about errors that happen in this unit | 27 (4.2%) | 34 (5.3%) |
| 18 | When errors happen in this unit, we discuss ways to prevent them from happening again | 24 (3.7%) | 36 (5.6%) |
| 19 | In this unit, we are informed about changes that are made based on event reports | 60 (9.3%) | 35 (5.4%) |
| 20 | In this unit, staff speak up if they see something that may negatively affect patient care | 20 (3.1%) | 35 (5.4%) |
| 21 | When staff in this unit see someone with more authority doing something unsafe for patients, they speak up | 63 (9.8%) | 35 (5.4%) |
| 22 | When staff in this unit speak up, those with more authority are open to their patient safety concerns | 110 (17.1%) | 37 (5.7%) |
| 23 | In this unit, staff are afraid to ask questions when something does not seem right | 36 (5.6%) | 41 (6.4%) |
| 24 | When a mistake is caught and corrected before reaching the patient, how often is this reported? | 206 (31.9%) | 34 (5.3%) |
| 25 | When a mistake reaches the patient and could have harmed the patient, but did not, how often is this reported? | 184 (28.5%) | 36 (5.6%) |
| 26 | In the past 12 months, how many patient safety events have you reported? | N/A | 34 (5.3%) |
| 27 | How would you rate your unit/work area on patient safety? | N/A | 35 (5.4%) |
| 28 | The actions of hospital management show that patient safety is a top priority | 119 (18.4%) | 33 (5.1%) |
| 29 | Hospital management provides adequate resources to improve patient safety | 106 (16.4%) | 35 (5.4%) |
| 30 | Hospital management seems interested in patient safety only after an adverse event happens | 142 (22.0%) | 34 (5.3%) |
| 31 | When transferring patients from one unit to another, important information is often left out^1^ | 48 (7.4%) | 32 (5.0%) |
| 32 | During shift changes, important patient care information is often left out | 16 (2.5%) | 36 (5.6%) |
| 33 | During shift changes, there is adequate time to exchange all key patient care information | 20 (3.1%) | 36 (5.6%) |

**Table S5.** Missing data for the Organizational Readiness for Change scale (ORC-SWE-S)

|  | N=645 | Missing data |
| --- | --- | --- |
| 1 | My organization needs guidance in evaluating staff performance | 17 (2.6%) |
| 2 | My organization needs guidance in improving relations among staff | 17 (2.6%) |
| 3 | My organization needs guidance in improving communications among staff | 14 (2.2%) |
| 4 | Current pressure to make changes comes from my manager/managers | 14 (2.2%) |
| 5 | Current pressure to make changes comes from hospital management | 17 (2.6%) |
| 6 | Current pressure to make changes comes from politicians | 18 (2.8%) |
| 7 | My workplace has problems with high staff turnover | 16 (2.5%) |
| 8 | Staff at my workplace usually have enough time to do their work | 13 (2.0%) |
| 9 | My workplace has enough staff to meet the patients' needs | 16 (2.5%) |
| 10 | I frequently share my knowledge and ideas with others | 23 (3.6%) |
| 11 | I am considered an experienced source of advice in my field | 0 (0.0%) |
| 12 | Other staff often ask for my advice | 19 (2.9%) |
| 13 | I am viewed as a leader by the staff I work with | 18 (2.8%) |
| 14 | Learning and using new procedures and routines is easy for me | 19 (2.9%) |
| 15 | I have a positive impact on my colleagues | 19 (2.9%) |
| 16 | I feel appreciated for the work I do | 24 (3.7%) |
| 17 | I highly value my contribution at work | 30 (4.7%) |
| 18 | I enjoy my work | 28 (4.3%) |
| 19 | Clinical equipment at my workplace is adequate (for example CTG monitors, ultrasound or other medical-technical equipment) | 20 (3.1%) |
| 20 | My work facilities are adequate | 21 (3.3%) |
| 21 | Computer and IT problems are usually repaired promptly at my workplace | 22 (3.4%) |
| 22 | Problems with clinical equipment are usually repaired promptly at my workplace | 24 (3.7%) |
| 23 | The staff are satisfied with our computer systems | 21 (3.3%) |
| 24 | Our organization has clear and well-defined goals | 23 (3.6%) |
| 25 | Management has a clear plan for how we are to achieve our goals | 23 (3.6%) |
| 26 | There is too much conflict between different professions | 28 (4.3%) |
| 27 | Staff have significant autonomy to make independent decisions at work | 26 (4.0%) |
| 28 | Staff have the autonomy to try out different ideas or ways of working | 25 (3.9%) |
| 29 | Managers are receptive to ideas and suggestions from the staff | 24 (3.7%) |
| 30 | Staff often show symptoms of stress or burn out | 25 (3.9%) |
| 31 | I have too much workload to do a good job | 25 (3.9%) |
| 32 | Changing routines to match new requirements is straightforward | 26 (4.0%) |
| 33 | Management makes well-informed decisions | 26 (4.0%) |

.

| 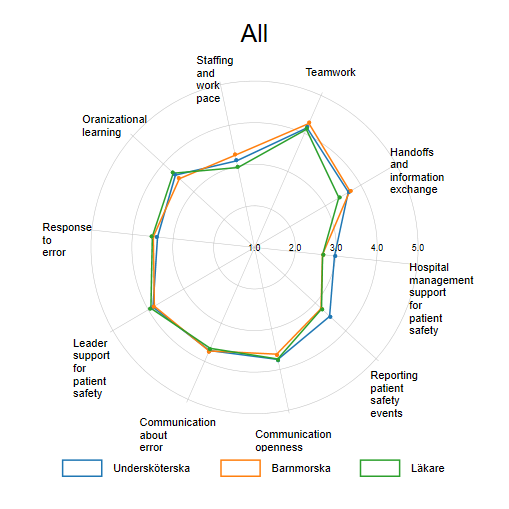  Nurse assistants  Midwives  Physicians  HSOPS 2.0  All labor wards (organizations) | 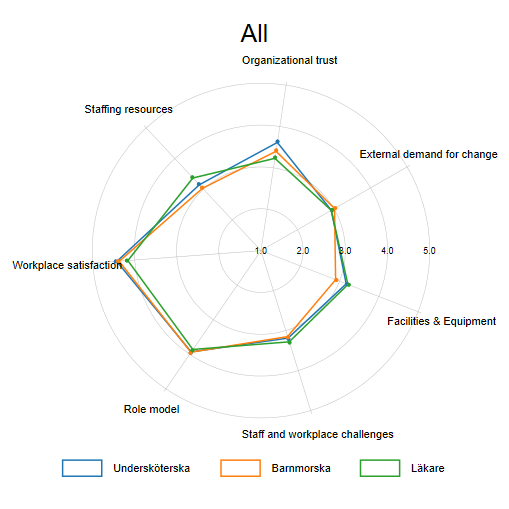  Midwives  Physicians  Nurse assistants  ORC-SWE-S  All labor wards (organizations) |
| --- | --- |

**Figure S2.** Comparison of the average response scores for each dimension of the Hospital Survey on Patient Safety Culture (HSOPS 2.0) and the Swedish shortened version of the Organizational Readiness for Change scale (ORC-SWE-S) categorized by profession.

| 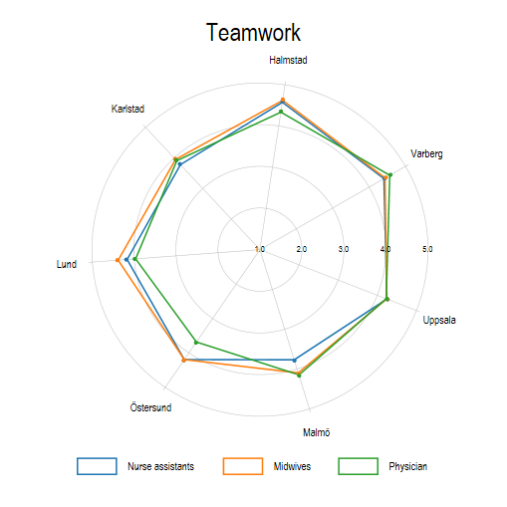  **A**  **C**  **D**  **G**  **B**  **E**  **F** | 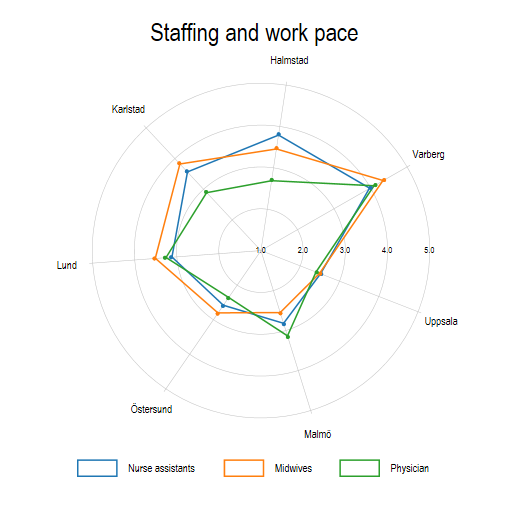  **A**  **C**  **F**  **E**  **D**  **B**  **G** | 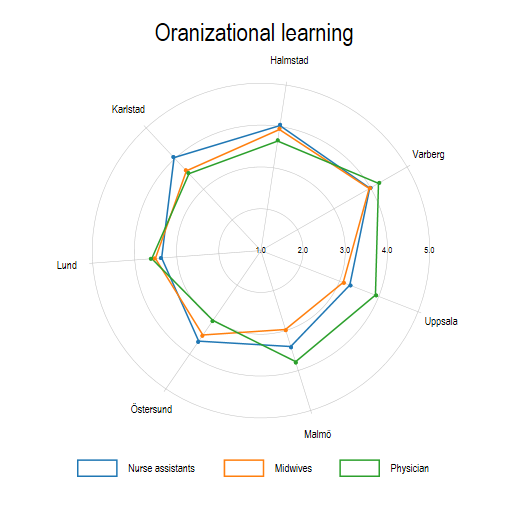  **A**  **C**  **F**  **E**  **D**  **B**  **G** | 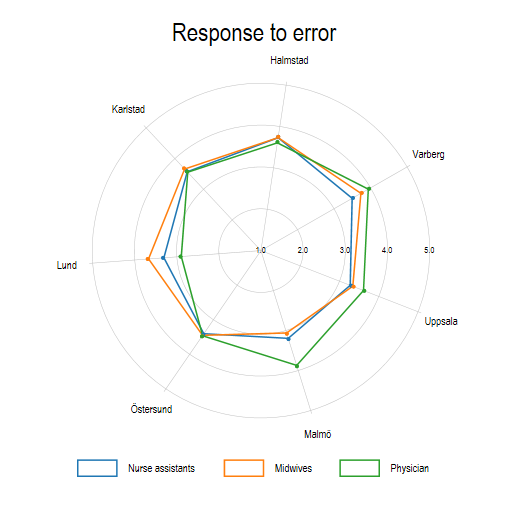  **A**  **C**  **F**  **E**  **D**  **B**  **G** |
| --- | --- | --- | --- |
| 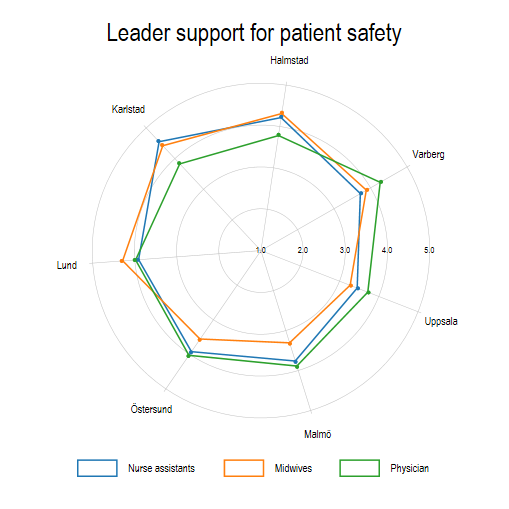  **A**  **C**  **F**  **E**  **D**  **B**  **G** | **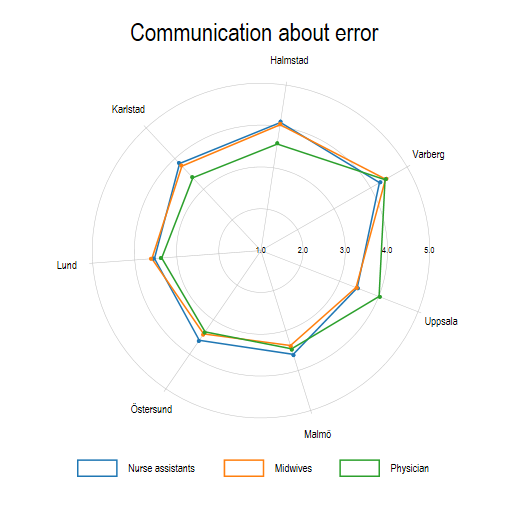**  **A**  **C**  **F**  **E**  **D**  **B**  **G** | 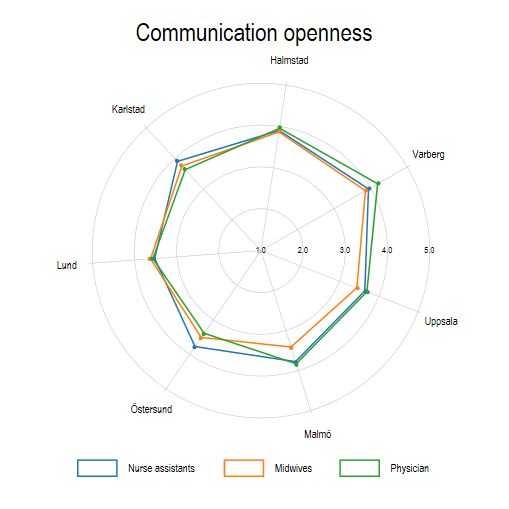  **A**  **C**  **F**  **E**  **D**  **B**  **G** | 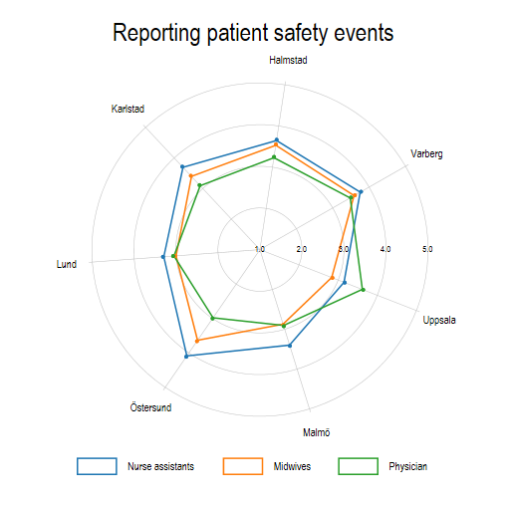  **A**  **C**  **F**  **E**  **D**  **B**  **G** |
|  |  |  |  |
|  |  |  |  |
|  |  |  |  |
|  |  |  |  |
|  |  |  |  |
| 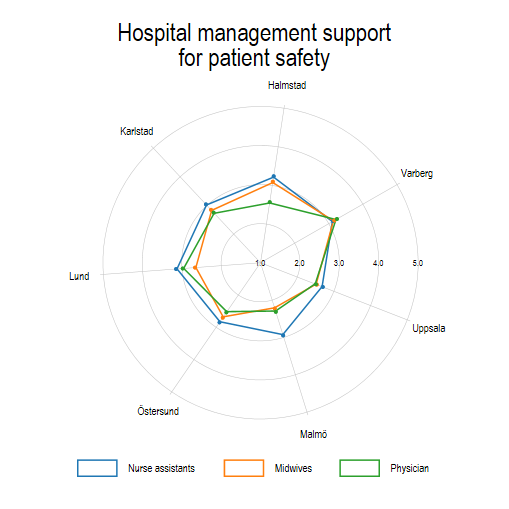  **A**  **G**  **D**  **F**  **C**  **B**  **E** | 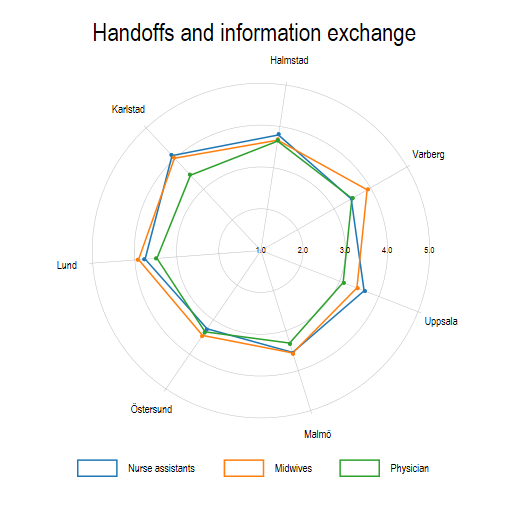  **E**  **G**  **D**  **F**  **C**  **B**  **A** |  |  |

Abbreviations for the labor wards: A: Region Southern Labor ward A, B: Region Southern Labor ward B, C: Region Central Labor ward C, D: Region West-Central Labor ward D, E: Region Southwestern Labor ward E, F: Region Southwestern Labor ward F, G: Region North-Central Labor ward G

**Figure S3.** Comparison of the average response scores across organizations for each dimension of the Hospital Survey on Patient Safety Culture (HSOPS 2.0), categorized by profession.

**A**

Role Model

Workplace Satisfaction

Inadequate Staffing Resources

Organizational trust

| 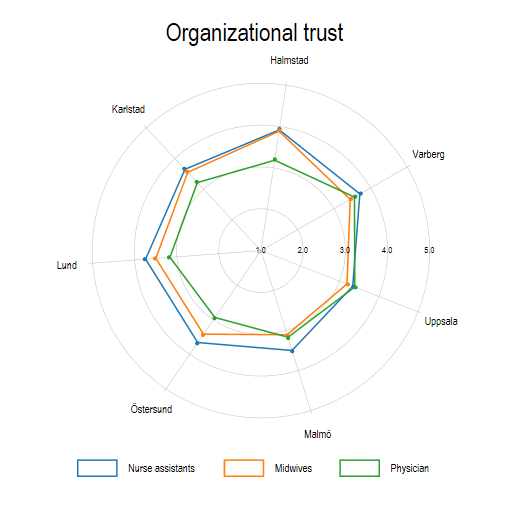  **G**  **F**  **E**  **D**  **C**  **B**  **A** | 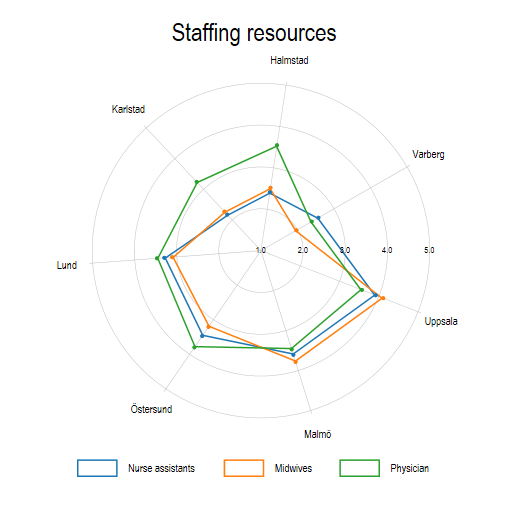  **G**  **F**  **E**  **D**  **C**  **B**  **A** | 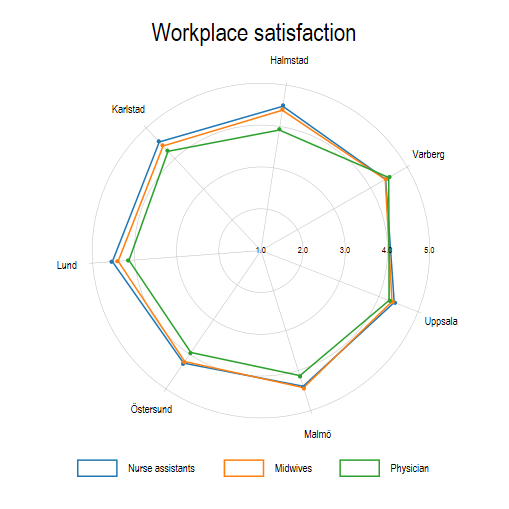  **G**  **F**  **E**  **D**  **C**  **B**  **A** | 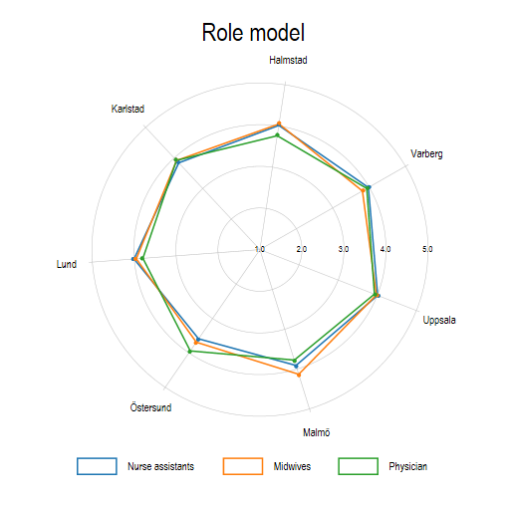  **G**  **F**  **E**  **D**  **C**  **B**  **A** |
| --- | --- | --- | --- |
| Staff and Workplace Challenges  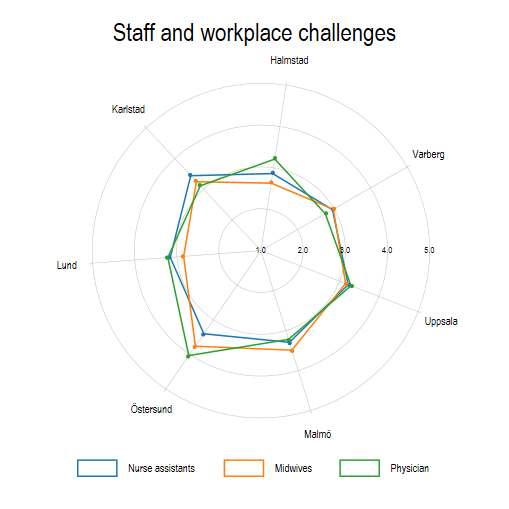  **G**  **F**  **E**  **D**  **C**  **B**  **A** | Facilities and Equipment  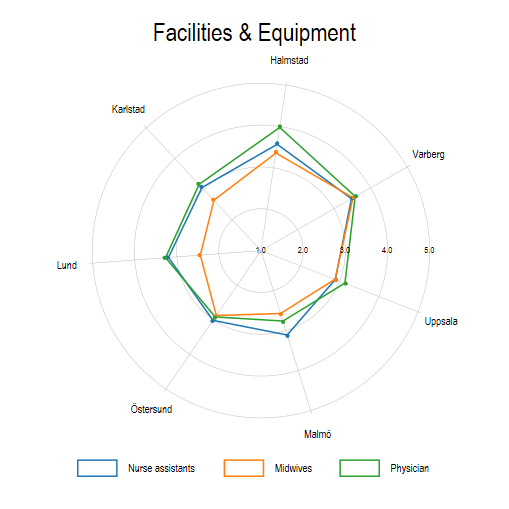  **G**  **F**  **E**  **D**  **C**  **B**  **A** | External Demand for Change  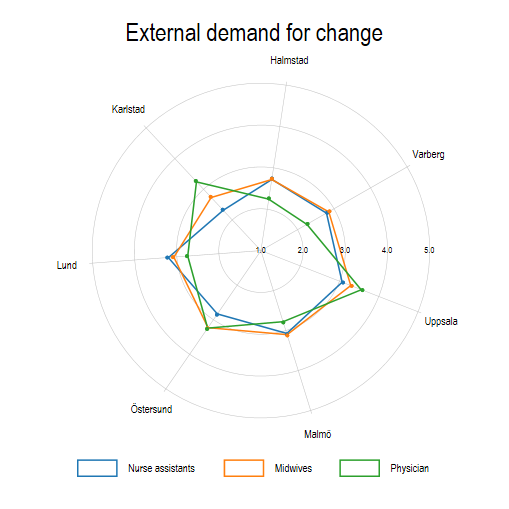  **G**  **F**  **E**  **D**  **C**  **B**  **A** |  |

Abbreviations for the labor wards: A: Region Southern Labor ward A, B: Region Southern Labor ward B, C: Region Central Labor ward C, D: Region West-Central Labor ward D, E: Region Southwestern Labor ward E, F: Region Southwestern Labor ward F, G: Region North-Central Labor ward G

**Figure S4.** Comparison of the average response scores for each dimension of the Swedish shortened version of the Organizational Readiness for Change scale (ORC-SWE-S) across organizations, categorized by profession.
